# Supplementary material for: An Intrinsic Role of Beta Oscillations in Memory for Time Estimation
Source: Sci Rep. 2018 May 22;8:7992. doi: 10.1038/s41598-018-26385-6 (PMC5964239; doi:10.1038/s41598-018-26385-6)
Supplement: Supplementary file 1 — Supplementary Material [file 41598_2018_26385_MOESM1_ESM.docx]

Supplementary Material for

**An Intrinsic Role of Beta Oscillations in Time Estimation**

Martin Wiener^1^, Alomi Parikh^2^, Arielle Krakow^3^, H. Branch Coslett^2^

^1^George Mason University ^2^University of Pennsylvania^3^Johns Hopkins University

Correspondence

Martin Wiener

Department of Psychology

George Mason University

Fairfax, VA

**Model Fitting and Testing.**

To account for model convergence, several steps were taken. First, model comparisons were conducted between models of varying complexity. We sought to compare a factorial expansion of our experimental design, and so we compared performance of an “empty” model, in which all model parameters varied only by subject, to three models of increasing complexity (frequency, frequency x stimulation, frequency x stimulation x duration). Model comparison was conducted using the deviance information criterion (DIC; (Spiegelhalter, et al. 2002); a DIC difference greater than 10 was taken as evidence in favor of a more complex model. Each model was estimated using a chain of 10,000 samples from the posterior distribution, with the first 1000 samples discarded as burn-in, and only every 5^th^ sample thereafter retained; this process, known as “thinning”, is used to increase chain stability and reduce autocorrelations between subsequent samples. Once the “winning” model was found, we further sampled five additional chains of 5000 iterations (200 burn-in) and compared the Gelman-Rubin Statistic (Gelman & Rubin, 1992). Finally, we conducted posterior predictive checks (PPC; (Krushke, 2014)) by using model parameters to simulate data from 500 observers and compare those data to our original set of subjects.

One aspect of the modeling approach here is that the parameters for each subject, in each condition, are estimated hierarchically, such that the group distribution is used to constrain values for individual subject and trial-wise parameters. We therefore sought to determine if this approach influenced our results. To do this, we also estimated DDM parameters in a “non-hierarchical” fashion, by applying our HDDM model to each subject individually, and then combining model values. We again compared factorial model complexity, to ensure that the winning, hierarchical model was not driven by the method of fitting. In this case, we compared the average Akaike Information Criterion (AIC) score between models, as the DIC was developed the assess hierarchical models only; further, AIC models can be compared by measuring the relative likelihood of each model compared to the model with the lowest AIC value (Burnham & Anderson, 2002).

Lastly, we also explored the application of a Hierarchical Generalized Linear Model (GLM) approach to HDDM, by applying the HDDMRegressor class of the HDDM toolbox. Here, the involvement of model parameters can be directly regressed onto choice and reaction time data. This approach allows for the direct comparison, via Bayesian statistics, of different posterior distributions. Again, model complexity was explored to pick the winning model (via DIC). In this case, the frequency of stimulation and stimulation status (baseline vs stim) were modeled as dummy variables, with [alpha,baseline] as the intercept in the most complex model. Duration was modeled as a continuous covariate. Parameters for *v, t,* and *a* were modeled as linear relations, whereas *z* was modeled by an inverse logit link function (see <http://ski.clps.brown.edu/hddm_docs/tutorial_regression_stimcoding.html#chap-tutorial-hddm-regression> for details on this process) to constrain parameter values between 0 and 1. Comparisons were done on the group posterior distributions to assess significance (Kruschke, 2014).

While the results above highlighted a number of distinct effects for tACS, including a differential effect on the starting point parameter between alpha and beta stimulation, we note that the effects observed may have been driven by the method used for fitting our model to the data. Specifically, we initially employed a hierarchical design, in which the individual subject posterior estimates are constrained by the group distribution (Kruschke, 2014). As such, our findings may have been driven by the non-independent nature by which they were sampled. To assess this, we conducted a separate, non-hierarchical sampling procedure, wherein each subject was modeled separately, and individual parameters estimates were combined afterwards. Comparison of the non-hierarchical model to the hierarchical one demonstrated relatively good concordance between both fitting methods (supplementary figure 6a), with the same pattern observed between stimulation and frequency-of-stimulation conditions. The only exception was for the threshold parameter, in which the non-hierarchical method did not reveal a decrease in the threshold for alpha tACS, suggesting caution in interpreting this result, a point we turn to below.

Given the discrepancy between hierarchical and non-hierarchical methods with regards to the threshold parameter for alpha stimulation, we sought to apply a hierarchical regression method to our data. Specifically, the hierarchical method we employed utilized separate nodes for each condition and each subject, and does not take into account within-subject covariance across conditions. To provide greater specificity of our model, we utilized the HDDMRegressor class of the HDDM toolbox to construct a hierarchical linear model. To accomplish this, each model DDM parameter was described by a general linear model with a random intercept for each subject, frequency (alpha, beta) and stimulation (pre-stim, stim) conditions as dummy variables, and duration as a continuous covariate that interacted with frequency and stimulation.

The results of the regression analysis are displayed in supplementary figure 6b. In general, the regression findings accord with the hierarchical and non-hierarchical findings. For the threshold parameter, we observed that slope value for stimulation was negative and did not fall within a region of practical equivalence (ROPE; (Kruschke, 2011)) of [-0.1 – 0.1] (β= -0.4476, 95% Credible Interval [-0.5166, -0.3822]), indicating that threshold values decreased with stimulation, whereas the interaction effect between stimulation and frequency encompassed the ROPE (β= 0.0669 95% CrI [-0.0215, 0.1594]), indicating that threshold values decreased with stimulation, but this effect did not vary by stimulation frequency. For the drift parameter, the stimulation slope was positive and was not within the ROPE (β= 8.2038, 95% CrI [8.0231, 8.3818]), indicating that drift rates overall increased with stimulation; notably, the interaction effect between stimulation and frequency was also positive and was not within the ROPE (β= 0.3821, 95% CrI [0.1418, 0.6399]), indicating that the increase in drift values was differential between alpha and beta frequency. For non-decision time, the stimulation slope was negative and did fall within the ROPE (β= -0.0489, 95% CrI [-0.0537, -0.0384]), as did the interaction (β= -0.0156, 95% CrI [-0.0268, -0.01]) indicating that non-decision time decreased with stimulation, but this effect could not be reliably differentiated from a null effect. Finally, for the starting point parameter, the stimulation slope was negative and fell within the ROPE (β= -0.1373, 95% CrI [-0.2336, -0.04]), whereas the interaction effect was positive and did not fall within the ROPE (β= 0.2552, 95% CrI [0.1102, 0.4145]), indicating there was no main effect of stimulation, but that stimulation and frequency interacted.

Overall, the results of the regression analysis corroborated the findings of the hierarchical and non-hierarchical fitting methods. We choose to rely on findings that agree across all three fitting methods when describing our results. Specifically, tACS, regardless of frequency, led to a decrease in the threshold and an increase in the drift parameters. For the non-decision time parameter, no effect of stimulation or interaction with frequency was observed. Finally, for the starting point parameter, no main effect of stimulation was observed, but an interaction with frequency was found, wherein the starting point shifted positively, towards the long duration boundary, for beta stimulation.

**References**

Burnham KP, Anderson DR. Information and likelihood theory: a basis for model selection and inference. Model Selection and Multimodel Inference: A Practical Information-Theoretic Approach.:49-97.

Gelman A, Rubin DB. Inference from iterative simulation using multiple sequences. Statistical science. 1992 Nov 1:457-72.

Kruschke JK. Bayesian assessment of null values via parameter estimation and model comparison. Perspectives on Psychological Science. 2011 May;6(3):299-312.

Kruschke J. Doing Bayesian data analysis: A tutorial with R, JAGS, and Stan. Academic Press; 2014 Nov 11.

Spiegelhalter DJ, Best NG, Carlin BP, Van Der Linde A. Bayesian measures of model complexity and fit. Journal of the Royal Statistical Society: Series B (Statistical Methodology). 2002 Oct 1;64(4):583-639.

**Supplementary Figures**


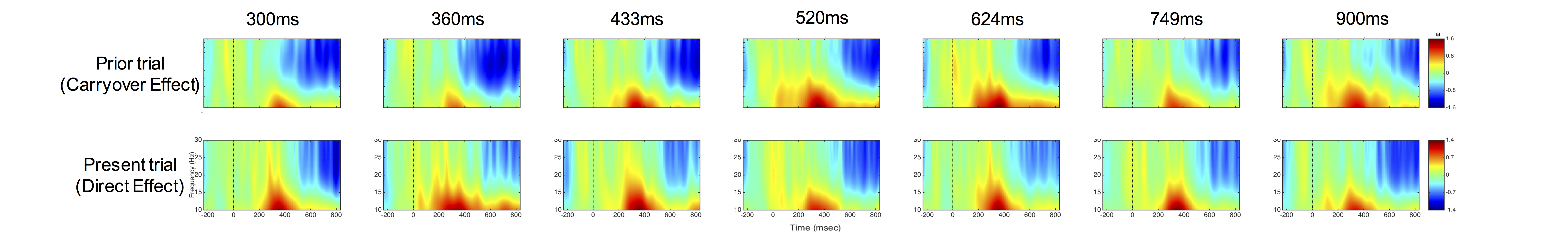


**Supplementary Figure 1 –** Direct and Carryover effects re-analysis from Wiener & Thompson (2015). Time/frequency plots of onset-locked responses to each of the seven durations presented on the current trial or prior trial (electrode FCz).

**Supplementary Figure 2 -** Number of trials for each interval across stimulation conditions after removal of trials where the reaction time exceeded 1000ms. A repeated measures ANOVA found a significant main effect of stimulation [*F*(1,18) = 5.107, p = 0.036, η^2^_p_ = 0.221], and a frequency by duration interaction [*F*(1,18) = 2.917, p = 0.021, η^2^_p_ = 0.139]. Specifically, fewer trials were filtered out for the beta stimulation session than for the alpha stimulation session. Due to these differences in trial count, we included the average number of trials per subject as a covariate in subsequent analyses of the bisection point, where the number of trials can impact the shape of the distribution (Fründ, et al. 2011). Shaded regions represent +/- standard error.

**Supplementary Figure 3 -** Carryover analysis of psychometric data (Wiener, et al. 2014). Top graphs display the mean perceptual asymmetry and decision bias values, respectively. Perceptual asymmetry reflects the influence of the preceding trial duration on the present trial duration; negative values indicate assimilation whereas positive values indicate repulsion. Decision bias reflects the influence of the preceding trial decision on the present trial decision; negative values indicate subjects are more likely to respond on each trial with the response made on the previous one. Bottom scatterplot displayed individual subject data points, demonstrating a correlation between both types of carryover, replicating prior findings (Wiener, et al. 2014). No significant effect of stimulation or frequency was observed for any carryover measure. We note that the removal of outliers from the above graph did not change the overall findings.

**Supplementary Figure 4 -** Fitting results of the different models used. DIC values are used for hierarchical and regression models, whereas the average AIC value is used for non-hierarchical fitting. In all cases, improved fits were found with more complex models, with the best fit for the Stimulation + Frequency + Duration model. A fifth model was also tested hierarchically, in which the variability of the drift rate (sv) was allowed to vary across duration (Balci & Simen, 2014). However, this model did not improve the fit over the best fitting model (ΔDIC <10).

**Supplementary Figure 5 -** Results of posterior predictive checks. Model simulations were run, using the peak parameters from the hierarchical DDM, to generate 500 “subjects”. Average model simulated values are plotted here for psychometric and chronometric data (filled lines) over average subject performance (closed points). In general, model simulations produced the same observed pattern in the observed data, with faster RTs for stimulation conditions, and a more pronounced leftward shift for beta stimulation.

**Supplementary Figure 6 -** Model fitting results summary. A) Results for hierarchical and non-hierarchical fitting methods, collapsed across duration, for all four parameters in the model (*a,v,t,z*). In general, individual and hierarchical fits agreed. The only exception is for threshold values, where individual fits suggest an interaction between stimulation and threshold. For the starting point, although pre-stimulation values differ, individual and hierarchical methods both suggest an interaction. B) Results of the regression analysis. Displayed are the posterior probability density distributions for each parameter value. Blue distributions represent the main effect of stimulation, whereas gold distributions present the interaction between stimulation and frequency. Dashed lines indicate the ROPE; distributions where the highest density region (95% credible interval) falls within this region cannot be reliably distinguished from a null effect. For non-decision time, both distributions fall within the ROPE.

**Supplementary Table 1 -** Sensation questionnaire responses during and after tACS. Bold points represent the average score, with italicized scores representing the standard error. Subjects were asked to rate each sensation on a 0 (no sensation) to 10 (max sensation) scale.
